# SMAD4 Loss triggers the phenotypic changes of pancreatic ductal adenocarcinoma cells

**DOI:** 10.1186/1471-2407-14-181

**Published:** 2014-03-14

**Authors:** Yu-Wen Chen, Pi-Jung Hsiao, Ching-Chieh Weng, Kung-Kai Kuo, Tzu-Lei Kuo, Deng-Chyang Wu, Wen-Chun Hung, Kuang-Hung Cheng

**Affiliations:** 1 Institute of Biomedical Sciences, National Sun Yat-Sen University, Kaohsiung 80424, Taiwan; 2 Division of Endocrinology and Metabolism, Kaohsiung Medical University Hospital, Kaohsiung, Taiwan; 3 Division of Hepatobiliary Pancreatic Surgery, Department of Surgery, Kaohsiung Medical University, Kaohsiung, Taiwan; 4 Division of Internal Medicine, Kaohsiung Municipal Hsiao-Kang Hospital, Kaohsiung Medical University, Kaohsiung, Taiwan; 5 Division of Gastroenterology, Department of Internal Medicine, Kaohsiung Medical University Hospital, Kaohsiung, Taiwan; 6 National Institute of Cancer Research, National Health Research Institutes, Tainan 704, Taiwan

**Keywords:** SMAD4/DPC4, TGFβ1 pathway, CD133, EGFR, Pancreatic cancer

## Abstract

**Background:**

SMAD4 is a gastrointestinal malignancy-specific tumor suppressor gene found mutated in one third of colorectal cancer specimens and half of pancreatic tumors. SMAD4 inactivation by allelic deletion or intragenic mutation mainly occurs in the late stage of human pancreatic ductal adenocarcinoma (PDAC). Various studies have proposed potential SMAD4-mediated anti-tumor effects in human malignancy; however, the relevance of SMAD4 in the PDAC molecular phenotype has not yet been fully characterized.

**Methods:**

The AsPC-1, CFPAC-1 and PANC-1 human PDAC cell lines were used. The restoration or knockdown of SMAD4 expression in PDAC cells were confirmed by western blotting, luciferase reporter and immunofluorescence assays. In vitro cell proliferation, xenograft, wound healing, quantitative reverse transcriptase-polymerase chain reaction (qRT-PCR), Western blotting, and immunohistochemistry analysis were conducted using PDAC cells in which SMAD4 was either overexpressed or knocked down.

**Results:**

Here, we report that re-expression of SMAD4 in SMAD4-null PDAC cells does not affect tumor cell growth *in vitro* or *in vivo*, but significantly enhances cells migration *in vitro*. SMAD4 restoration transcriptionally activates the TGF-β1/Nestin pathway and induces expression of several transcriptional factors. In contrast, SMAD4 loss in PDAC leads to increased expression of E-cadherin, vascular endothelial growth factor (VEGF), epidermal growth factor receptor (EGFR) and CD133. Furthermore, SMAD4 loss causes alterations to multiple kinase pathways (particularly the phosphorylated ERK/p38/Akt pathways), and increases chemoresistance *in vitro.* Finally, PDAC cells with intact SMAD4 are more sensitive to TGF-β1 inhibitor treatment to reduced cell migration; PDAC cells lacking SMAD4 showed decreased cell motility in response to EGFR inhibitor treatment.

**Conclusions:**

This study revealed the molecular basis for SMAD4-dependent differences in PDAC with the aim of identifying the subset of patients likely to respond to therapies targeting the TGF-β or EGFR signaling pathways and of identifying potential therapeutic interventions for PDAC patients with SMAD4 defects.

## Background

Pancreatic cancer is one of the most insidious forms of human cancer whose incidence nearly equals its death rate. Histologically, ductal adenocarcinomas of the pancreas (PDAC) account for > 90% of all exocrine pancreatic cancers. PDAC remains the eighth leading cause of cancer death worldwide, with the lowest 5-year survival rate of any gastrointestinal cancer. Several features conspire to make PDAC a formidable clinical issue: poor early detection, the advanced nature of most tumors at the time of diagnosis, and lack of specific or effective therapy. In contrast to other major cancers, decades of clinical trials have failed to provide appreciable survival and less toxicity benefit for PDAC [1]. For example, FOLFIRINOX and nab-Paclitaxel for treatment of advanced pancreatic cancer have shown to be effective for overall survival, progression-free survival, and response rate, but was associated with increased toxicity and serious side effects [2-4]. Indeed, this continual cycle of clinical trial for PDAC therapy followed by failure has led some to conclude that there is insufficient knowledge of the mechanisms underlying this particular type of lethal disease [5,6].

A number of studies of PDAC have elucidated a detailed profile of genetic alterations associated with PDAC initiation and progression — including the activation KRAS and loss of INK4A, p53, and SMAD4 — providing clues for investigation of the molecular and biochemical basis for this malignancy [7,8]. SMAD4 is recognized as an intracellular common mediator for the TGF-β superfamily signaling pathways, including TGF-β1, activin, and BMP signaling, responsible for embryonic patterning, differentiation and a variety of homeostatic processes [9,10]. During the initiation phase of carcinogenesis, most malignant epithelial tumors develop resistance to TGF-β/SMAD-mediated growth inhibition. However, excessive levels of TGF-β1 are associated with malignant tumor progression in many cancers, suggesting that inactivation of the SMAD proteins could be an important event in this process [11]. With respect to cellular growth control, the effects of TGF-β are highly dependent on the cell type and cell context, which exert alternating growth-promoting and growth-inhibitory effects in different cell types and at different stages of tumorigenesis. Several independent studies indicate that deletions or intragenic mutations of the SMAD4 gene are present in more than 50% of human PDACs, but are rare in other malignancies such as lung or breast cancer [12-16]. Hence, SMAD4 is a distinguishing molecular feature of two major types of PDAC. Although many lines of evidence indicate that SMAD4 status in PDAC is associated with specific histopathological phenotypes, the detailed molecular basis of SMAD4-dependent phenotypic changes in cancer biology has yet to be determined.

Although many lines of evidence indicate that inactivation of SMAD4 in PDAC is generally restricted to high grade Pancreatic intraepithelial neoplasia (PanIN) and PDAC, implying a specific role for SMAD4 in malignant progression, the specific anti-tumorigenic impact of SMAD4 loss has not been fully characterized [8,17]. Notably, studies of human cell lines have given inconsistent results of how SMAD4 status influences TGF-β responsiveness and of other tumor biological properties, leading to conflicting conclusions on the impact of SMAD4 defects on PDAC prognosis [18,19]. Overall, these studies suggest that TGF-β/SMAD4 signaling may have pleiotropic and context-dependent roles during PDAC progression. These features add significant complexity to attempts to design therapeutic strategies to deregulate the SMAD4 pathway. In this study, we used SMAD4-proficient and -deficient human PDAC cell lines AsPC-1, CFPAC-1, and PANC-1 to compare the molecular profiles of SMAD4-positive and -negative PDAC cells; assess their relationship to SMAD4 status; and further demonstrate the ability of SMAD4 to modulate cell proliferation, affect cell motility, regulate the epithelial-mesenchymal-transition (EMT) process, activate kinase pathways, change expression of cancer stem-like cell (CSC) markers and affect sensitivity to chemodrugs in PDAC. The objective of the present study was therefore to dissect the molecular circuits that contribute to the inactivation of SMAD4 in different phenotypes of PDAC.

## Methods

### Cell culture, RNA isolation, and cDNA synthesis and inhibitors treatments

The HEK293T and human PDAC cell lines were obtained from sources described previously [8,20]. Treatments with TGF-β1 (5 ng/ml), cisplatin, paciltaxol, gemcitabine, SB231542 and gefitinib were performed according to previously-described procedures [20,21]. The RNA isolation and cDNA synthesis from the cell lines were also conducted according to previously-described protocols [20,22].

### Plasmid and retroviral construction

A full length cDNA clone for the SMAD4 gene was originally obtained from the Bert Vogelstein laboratory and subcloned in pBabe-puro plasmid (Addgene, Cambridge, MA) to create a pBabe-SMAD4-puro vector [21]. In brief, for SMAD4 gene restoration, pBabe-puro plasmid was digested with restriction enzyme BamHI and Hind ΙΙΙ to obtain the full length of SMAD4 cDNA, then ligated into BamHI/XhoI-digested pBabe-puro backbone vector. The insert fragment of SMAD4 cDNA was subcloned into the pBABE-puro backbone by using T4 ligase (NEB) subjected to Klenow enzyme reaction and ligated. All plasmids were verified by DNA sequencing (Genome International Biomedical Co., Ltd., New Taipei City, Taiwan).

### Retroviral production and infection of target cells

Retrovirus was generated by co-transfection of pBabe-puro empty vector or pBabe- puro-SMAD4 with pVSV-G (envelope) and pVSV-GP (packaging) plasmids in 293 T cells. Target cells were infected overnight with 4 ml of virus-containing medium in the presence of 10 μg/ml polybrene. The following day, cells were cultured in fresh medium and allowed to grow for another 24 hrs. After this medium was replaced with fresh regular medium, cells were selected with 2 μg/ml puromycin for 2 weeks. Positive stable clones were then characterized and utilized in further assays.

### Lentivirus production and shRNA for gene knockdown

All plasmids required for shRNA lentivirus production were purchased from the National RNAi Core Facility, Academia Sinica, Taipei, Taiwan. The pLKO.1-shRNA vector used for knockdown of SMAD4 was TRCN000010323 (SMAD4), and the scrambled lentiviral control vector was pLKO_TRC025. Lipofectamine 2000 reagent (Invitrogen, Carlsbad, CA) was used for lentiviral production in 293 T cells with a packaging construct (pCMV-ΔR8.91), an envelope construct (pMD.G) and different shRNA constructs as previously described [20].

### Western blotting

Western blotting was performed as described previously [20,21]. The following antibodies were used in this study: anti-SMAD4 (sc-7154 or sc-7966), anti-E-cadherin (sc-8426), anti-vimentin (sc-7557), anti-CD133 (sc-8304), anti-CD44 (sc-18849), anti-Sp1(sc-14027), anti-c-Jun (sc-1694), anti-Fos (sc-52), anti-Fast-1 (sc-377358), anti-Hes1 (sc-25392), anti-GAPDH (sc-32233; Santa Cruz Biotechnology, Inc.), anti-p-Akt (#4060), anti-Akt (#4691), anti-p-p44/42 (#9101),anti-p44/42 (#4695), anti-Pten (#9272), anti-NF-κB (#4764S), anti- EGFR (#4267), anti-p-EGFR tyr 992 (#2235), anti-p-EGFR tyr 1068 (#3777), anti-Smad2/3 (#5339), anti-p-Smad2/3 (#3101), anti-p-c-Jun (#2361; Cell Signaling Technology, Inc.), anti-Nestin (N5413), mouse anti-β-actin (Sigma- Aldrich Co.), anti-CD133/1 (AC133, Miltenyi Biotec.) and anti-TGF-β1 (ab9758, Abcam, Plc.).

### Quantitative reverse transcription polymerase Chain reaction (RT-qPCR) analysis

Total RNA prepared from samples was used for cDNA synthesis. PCR amplification and results of the delta computed tomography (CT) measurements were described previously [20,22]. The primers sequence used in thi stsudy were as follows: GAPDH primer sequences: forward 5′-GAAGGTGAAGGTCGGAGTCA-3′. Reverse 5′-AATGAAGGGGTCATTGAT GG-3′. SMAD4 primer pair: Forward 5′-CGCTTTTGTTTGGGTC AACT-3′. Reverse: 5′-CCCAAACATCACCTTCACCT-3. CD133 primer pair: Forward 5′-CCCCAGGAAATTT GAGGAAC-3′. Reverse 5′- TC CAACAATCCATTCCCTGT-3′. E-cadherin primer pair: Forward 5′-ATTGCAAATTCCTGCCATTC-3′. Reverse 5′-CTCTTCTCCGCCTCCTTCTT-3′. N-cadherin primer pair: Forward 5′-CCTTGTGCTGATGTTTGTGG-3′. Reverse 5′-TGGATGGGTCTTTCATCCAT-3′. vimentin primer pair: Forward 5′-GGGAGAAATTGC AGGAGGAG-3′. Reverse 5′-ATTCCACTTTGCGTTCAAGG-3′. CD44 primer pair: Forward 5′-AG ACACCATGCATGGTGCACC-3′. Reverse 5′-TAACAGCATCAGGAGTG-3′. EGFR primer pair: Forward 5′-TCAGCCACCCATATGTACCA-3′. Reverse: 5′-CATTC TTTCATCCCCCTGAA-3′. VEGF primer pair: Forward 5′-CCCACTGAGGAGTCC AACAT-3′. Reverse: 5′-T GCATTCACATTTGTTGTGC-3′. The PCR reactions were repeated three times from three independent experiments.

### Transient transfections and luciferase reporter assays

Transient transfections and SBE4 (four repeats of SMAD binding element), CD133 and Nestin luciferase reporter assays were performed as described previously [20].

### Cell proliferation assay

Cell proliferation assay was performed as previously described [20,22]. Briefly, 5X 10^3^ cells were seeded in 96-well plates, and incubated overnight. The cells were treated with or without drugs, and incubated for 1 to 3 days. 5 mg/ml MTT (thiazolyl blue tetrazolium bromide) (Americo Chemical Co) 25 μl in 500 μl medium was then added, and incubated for another 2 hours for reaction. The medium was removed, and crystal was completely dissolved with 200 μl DMSO (Sigma). The OD570 reading was then detected with a BioTek ELISA reader (Molecular Device, Sunnyvale, CA).

### *In vitro* cell migration/invasion assays

For wound healing cell migration assay, cells were pretreated with 0.02% (0.2 mg/mL) mitomycin C for 2 hours, and wounded by removing a 300–500 μm-wide strip of cells across the well with a standard 200 μL yellow tip. Wounded monolayers were washed twice with 1xPBS to remove nonadherent cells. The cells were cultured in low FBS media and incubated for pre-determined times to monitor wound closing. Wound closure was recorded by phase-contrast microscopy according to previously published protocols [20,22]. For transwell migration assays, 5 × 10^4^ cells were plated in the top chamber with a non-coated filter membrane (6-well insert, pore size 8 μm; BD Biosciences, San Jose, CA) in low serum medium. The bottom medium was supplemented with 10% FBS. Cells were incubated for 24 hours. Cells that did not migrate through the pores were removed by cotton swab. Cells on the lower surface of the membrane were stained with crystal violet before photography. The crystal violet was dissolved in 10% acetic acid and absorbance was measured by using the BioTek enzyme-linked immunosorbent assay (ELISA) reader OD570 (Level BioTek Instruments, Inc., Winooski, VT) for quantitative analysis [20].

### Mice and injections

To study *in vivo* tumorigenicity, pathogen-free female C.B17/lcr- SCID mice, eight weeks old, were purchased from BioLASCO Taiwan Co., Ltd. (Taipei, Taiwan). Technology from Charles River Laboratories (Wilmington, MA, USA) was used for breeding in the animal center at the Department of Medical Research, Kaohsiung Medical University (KMU) Hospital. Mice were housed at the Experimental Animal Center, KMU under specific pathogen-free (SPF) conditions under protocols approved by the KMU IACUC institutional guidelines for the care and use of experimental animals were followed. Mice were injected subcutaneously in the left and right flank with 1 × 10^6^ cells in 0.1 ml of medium. After two months, tumor volumes, overall health and total body weights of the mice were assessed as previously described [20]. Each experimental group contained > 4 mice.

### Mouse surgery, necropsy, histopathology and immunohistochemistry

Tissue samples were fixed in 10% buffered formalin for 12 h, washed with PBS and transferred to 70% ethanol, embedded in paraffin, sectioned and stained with hematoxylin and eosin (H&E). Immunohistochemical analysis of SMAD4, EGFR, E-cadherin, CD133 and Nestin were performed as described previously [8,20].

### Statistical analysis

Data are presented as mean ± standard error of the mean. The continuous data were statistically analyzed using Student’s *t*-test and categorical data were subjected to Chi-square test. All statistical calculations were performed using SAS for Windows version 12.2 (SAS, Inc., Cary, NC). A *p* value of less than 0.05 was considered significant [20].

## Results

### Generated stable SMAD4 over-expression and knockdown of human PDAC cells

**Figure 1:**
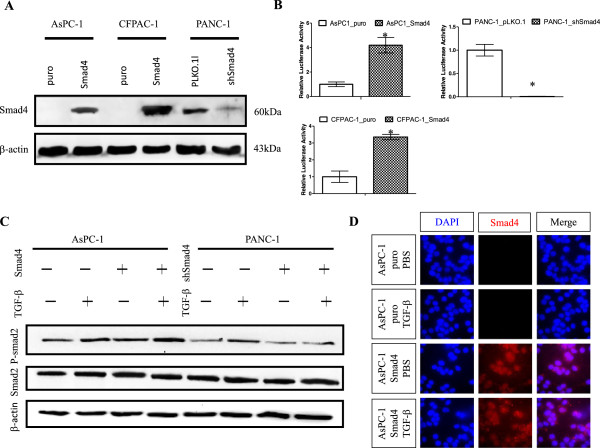
**Generated stable SMAD4 rerexpressing or knockdown of SMAD4 in human PDAC cells. (A)** Western blot analysis indicated that SMAD4 was successfully restored or knocked down in human PDAC cells as compared to control cells. β-actin was used as an internal control. **(B)** AsPC-1, CFPAC-1 and PANC-1 SMAD4 proficient and deficient cells were transiently transfected with SBE4 luciferase reporter and Renilla luciferase constructs. Cells were treated under the indicated conditions for 24 hours. Luciferase reporter assays were conducted using a dual luciferase assay, and Renilla luciferase activity was used as an internal control. Mean + SE (n = 3) *P <0.01. **(C)** Western blot detection of total and phosphorylated SMAD2 in SMAD4 proficient or deficient AsPC-1 and PANC-1 cells with or without TGF-β (10 ng/μl). Result confirmed TGF-β increased phosphorylation of SMAD2 in the SMAD4 restoration cell lines. β-actin was used as an internal control. **(D)** Immuno- fluorescence analysis confirmed that SMAD4 expression in AsPC-1 SMAD4 cells and mock control cells, and the nuclear localization of SMAD4 was observed in response to TGFβ1 treatment (magnification ×100).

To gain insight into the functional role of SMAD4 loss in PDAC cells, we first selected two SMAD4-deficient PDAC cell lines (AsPC-1 and CFPAC-1) and SMAD4 wild-type PANC-1 cells as the model cell lines in which to study the anti-tumor effects of SMAD4 in human PDAC. We generated the pBabe retrovirus construct expressing human SMAD4 to restore SMAD4 gene expression in SMAD4-deficient PDAC cell lines. To verify the restoration of SMAD4 in SMAD4-null AsPC-1 and CFPAC-1 cells, we first performed RT-qPCR analysis to examine the SMAD4 mRNA expression levels in those stable SMAD4 reconstituted PDAC cells; our results showed that the SMAD4 mRNA levels increased about 10-fold in comparison with puro control cells (data not shown). Western blotting analysis further confirmed the restoration of SMAD4 protein expression in the SMAD4-deficient PDAC cell lines AsPC-1, and CFPAC-1 (Figure 1A).

Further, we determined that the intact TGF-β signal pathway was fully restored in AsPC-1 and CFPAC-1 stable SMAD4 reconstituted cells by using a SBE4 luciferase reporter assay, and by detecting the levels of SMAD2 phosphorylation after TGF-β1 treatment in AsPC-1 cells after SMAD4 restoration (Figure 1B and C). We also observed that TGF-β1 treatment leads to nuclear translocation of SMAD4 in SMAD4-re-expressing AsPC-1 cells by immunofluorescence analysis (Figure 1D). Meanwhile, we utilized a shRNA-mediated RNA interference approach to knockdown the expression of SMAD4 in the PANC-1 cell line. Results of Western blots from the PANC-1 shSMAD4 cells showed a significant reduction of SMAD4 protein levels compared to mock control cells (Figure 1A). We also confirmed the reduced TGF-β1 signaling by phospho-SMAD2 western blot analysis and SBE4-luciferase activity assay in PANC-1 shSMAD4 cells when compared with control cells. (Figure 1B and C).

### SMAD4 restoration does not affect their proliferation *in vitro* and *in vivo*, but increases PDAC cells migration *in vitro*

**Figure 2:**
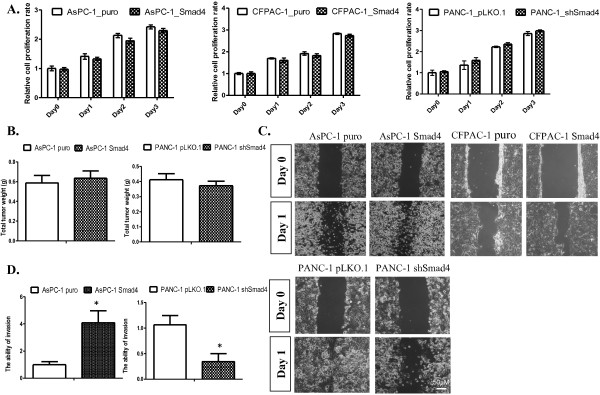
**SMAD4 does not significantly affect PDAC cell viability or proliferation, but increase PDAC cell motility in vitro. (A)** SMAD4 does not significantly affect growth of PDAC cells in vitro. Cells were seeded (5X10^3^ cells per well) in 96-well plates and cell proliferation rates were determined by MTT assay at indicated time points. **(B)** SMAD4 does not affect PDAC tumor growth in SCID mice. Xenograft tumors were established using SMAD4 proficient or deficient AsPC-1 and PANC-1 cells implanted by s.c injection (1 × 10^6^ cells) and analyzed after 8 weeks. Tumor weights were measured at autopsy. Mean + SE (n = 6). **(C)** Wound healing assays indicated that SMAD4 restoration reduces PDAC cells migration in vitro. The closure rates of cell free gap were recorded by phase contrast microscope after overnight incubation. Similar results were reproduced in three independent experiments. (magnification x40). **(D)** SMAD4 promotes the invasive ability of PDAC cells in vitro. Invaded cells were fixed and stained with crystal violet, and quantitative results were normalized against vector controls. Mean + SEM (n > 3). *p < 0.05.

Next, we explored the overall physiological effects of SMAD4 re-expression on PDAC cells *in vitro*. To determined if SMAD4 restoration has an effect on cell proliferation in SMAD4-deficient PDAC cells *in vitro*, we performed MTT assays in AsPC-1 and CFPAC-1 SMAD4 cells to determine the growth inhibitory effect, if any, of SMAD4. As shown in Figure 2A, our results indicated that SMAD4 restoration in AsPC-1 and CFPAC-1 cells did not significantly decrease the cell proliferation rate over that of the control cell lines following 3 days of normal cell culture condition. Thus, we concluded that SMAD4 restoration in most PDAC-deficient cell lines has a minimal effect on cell proliferation *in vitro*. Similarly, SMAD4 shRNA lentivirus-mediated stable knockdown for SMAD4 expression does not significantly affect cell growth in PANC-1 cells *in vitro* (Figure 2A). In addition, our *in vivo* study using subcutaneous xenografts in SCID mice revealed that SMAD4 re-expression in AsPC-1 cells or its knockdown in PANC-1 does not significantly affect tumor growth *in vivo* (Figure 2B).

To further investigate the effect of SMAD4 expression on the migratory potential of AsPC-1, CFPAC-1 and PANC-1 cells *in vitro*, *in vitro* wound healing assays were employed in SMAD4-proficient and -deficient CFPAC-1 and AsPC-1 cells. Monolayers of cells were pretreated with mytomycin-C for 2 hrs before being scratched with a pipette tip, and then cultured in the regular culture condition containing 5% fetal bovine serum (FBS). After overnight incubation, our results indicated that SMAD4 restoration significantly enhanced the ability *in vitro* of CFPAC-1 and AsPC-1 cells to migrate as compared to control cells (Figure 2C). In addition, knockdown of SMAD4 by shRNA significantly decreased the in vitro migratory potential of PANC-1 cells (P < 0.05; Figure 2C). Further, our results with *in vitro* invasion assay using a transwell chemotaxis invasion approach in AsPC-1 and PANC-1 cells also showed that SMAD4 enhanced the invasive ability of PDAC cells *in vitro* (P < 0.05; Figure 2D and Additional file 1: Figure S1).

### SMAD4 modulates EMT and regulates CSC-associated gene expression

**Figure 3:**
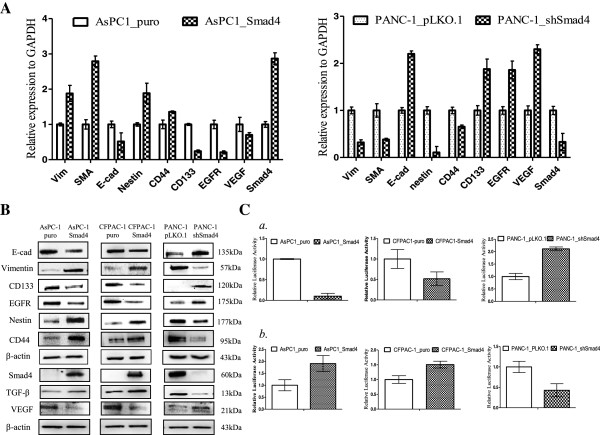
**SMAD4 reduces E-cadherin, VEGF, EGFR and CD133 expression, but increases TGFβ1/Nestin and CD44 protein levels in PDAC. (A)** SMAD4 modulates mRNA levels of EMT and CSC markers in PDAC cells. RT qPCR analyses were performed in AsPC-1 and PANC-1 SMAD4 deficient or proficient cells. Compared to the control, restoration of SMAD4 reduces mRNA level of EGFR, VEGF, CD133 and E-cadherin, but increased Vimentin, SMA, Nestin and CD44 mRNA expression in PDAC. Data were means ± SD of triplicates. *P < 0.05 **(B)** Western blot analysis of three PDAC cell lines, which had overexpression or knockdown for SMAD4 revealed that the expression levels of CD133, Nestin, EGFR, VEGF and EMT markers in indicated cell lines. β-actin was included as a loading control. **(C)** Luiferase activity assays for the analysis of CD133 and Nestin transcriptional activities in PDAC cells. Reproter assays were performed using CD133-luc (*a*) and Nestin-luc(*b)* reporter constructs in AsPC-1, CFPAC-1 and PANC-1 Smad4 proficient and deficient cells. Bars represent means derived from at least 3 independent experiments. *P < 0.01.

We and others have shown that SMAD4 is involved in regulating E-cadherin expression in PDAC [8]. One recent study also suggested that SMAD4 is required for TGF-β-induced EMT to mediate bone metastasis of breast cancer cells [23]. Thus, to further confirm that SMAD4 re-expression was involved in alterations of the EMT phenotype marker in PDAC, we performed RT-qPCR and Western blot analysis to evaluate the mRNA and protein levels of EMT-related markers in SMAD4-proficient and -deficient PDAC cells. As shown in Figure 3A, we observed up-regulation of smooth muscle actin and vimentin in the mRNA as well as protein levels and significantly lower levels of E-cadherin in SMAD4-proficient PDAC cells. Meanwhile, pancreatic CSC markers such as CD44, Nestin and CD133 have been shown to play important roles in maintaining PDAC progression. To assess whether SMAD4 re-expression induces alterations in the expression of these CSC markers in PDAC, we further determined the mRNA and protein expression levels of CD44, CD133 and Nestin on SMAD4-deficient and -proficient PDAC cells by RT-qPCR and Western blot analysis. Our Western blot analysis showed that SMAD4-proficient cells express more Nestin and CD44 proteins than SMAD4-deficient cells (Figure 3B). In contrast, the level of CD133 protein expression was reduced in the SMAD4-proficient cells compared to SMAD4- deficient cells (Figure 3B). Additional IHC analysis confirmed a significant increase of E-cadherin, EGFR and CD133 signals and reduced expression of Nestin protein in xenograft tumor samples belonging to PANC-1 shSMAD4 tumors as compared with the control group (Additional file 2: Figure S2). Meanwhile, luciferase reporter assays also confirmed transcriptional regulation of the CD133 and Nestin genes by SMAD4 in PDAC cells (Figure 3C(a and b)).

### Re-expression of SMAD4 reduces EGFR and VEGF expression and repression phosphorylation in the Akt and ERK signaling pathways, but enhances the p38 MAP kinase pathway

**Figure 4:**
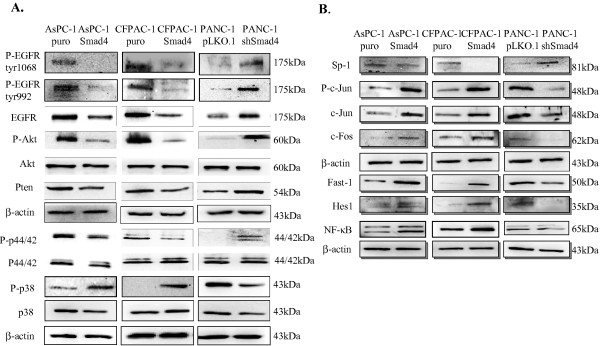
**SMAD4 modulates multiple kinase pathways and influences transcriptional factors expression. (A)** Restoration of SMAD4 results in a marked increase of p38 MAPK activation, but significantly attenuated the phosphorylation (activation) of EGFR and its downstream signals p44/42 (MEK) and Akt pathways in PDAC cells. **(B)** SMAD4 affects the expression transcriptional factors in PDAC cells. Total protein extracted from indicated cells were lysed for Western blot analysis. Western blots were then performed with the indicated antibodies. β-actin was served as an internal control.

SMAD4 has been shown to influence EGFR and VEGF expression in human normal pancreatic ductal cells (HPDEC) and Hs766T human pancreatic cancer cells [24,25]. To confirm these finding, cell lysates were collected from stably-SMAD4-expressing PDAC cells and control groups to examine the levels of VEGF and EGFR protein expression as well as phosphorylated EGFR by Western blot analysis. Western blot analysis revealed similar results in our PDAC cells to those of the previous studies. As shown in Figures 3B &4A, our Western blot analysis revealed that SMAD4 re-expression results in a decreased VEGF and EGFR protein levels. In addition, the reduced levels of EGFR leads to decreased EGFR phosphorylation levels at Y992 and T1068, and decreased phosphorylation of EGFR also elicits reduction of several downstream kinase pathways. The involvement of the ERK (p44/42) and Akt pathways in EGFR-dependent phosphorylation cascades is well recognized. Activation of the non-SMAD Akt and MAPK pathways, particularly p38 and p44/42 ERK, has been implicated in TGF-β1 signaling. To further determine the potential relationship of these kinase pathways to SMAD4 loss in PDAC cells, the levels of p-Akt, p-p44/42 and p-p38 were examined by Western blot analysis in SMAD4-reconstituted and vector-control PDAC cells. Western blot analysis revealed that the phosphorylation levels of p44/42 and Akt were both reduced in AsPC-1 and CFPAC-1 SMAD4-reconstituted cells, but phosphatase and tensin homolog (PTEN) protein expression was not increased in SMAD4 transfected cells compared to cells with the control vectors (Figure 4A), implying that SMAD4 loss not only increased the protein and phosphorylation levels of EGFR, but also activated the EGFR downstream signaling. We also observed that the re-expression of SMAD4 increased the phosphorylated and total levels of protein in the p38 MAP kinase pathway by Western blot analysis (Figure 4A). To confirm these findings, we used the shRNA strategy to compare PANC-1 cells with control shRNA; similar results were obtained (Figure 4A). These findings strongly suggest that re-expression of SMAD4 attenuates the Akt and Erk (p44/42) pathways and promotes p38 kinase activation in PDAC. Notably, in our Western blots to detect SMAD4-signaling-mediated effects on the expression of major transcriptional factors, we observed that SMAD4 elevated the expression of the transcriptional factors c-Jun, c-fos, Fast-1, Hes-1 and NF-κB but inhibited the expression of the transcriptional factors Sp-1 in PDAC cells (Figure 4B).

### SMAD4 defect confers chemoresistance and leads to augmented EGFR-mediated cancer cell motility in PDAC

**Figure 5:**
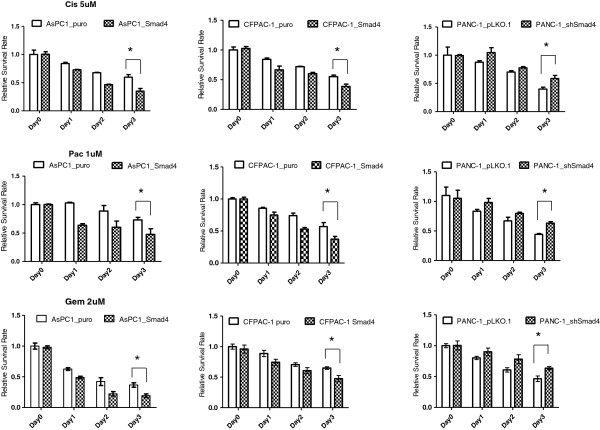
**SMAD4 loss contributes to chemoresistance of PDAC cells.** AsPC-1, CFPAC-1 and PANC-1, SMAD4 deficient and proficient cells were treated with different concentration of cisplatin (Cis; 5 μM), paciltaxol (Pac; 1 μM) and gemcitabine, (Gem; 2 μM) for 3 days. Chemosensitivity testing of PDAC cells using the MTT colorimetric assay. The data indicates SMAD4 increases drug sensitivity to three different chemo drugs in PDAC cells. Data were means ± SD of triplicates. *P < 0.01.

Since somatic inactivation of SMAD4 occurs primarily at later stages of pancreatic malignancy, and SMAD4 inactivation was reported to serve as a worse prognostic factor in PDAC patients who received adjuvant radiotherapy and chemotherapy, we next investigated whether restoration of SMAD4 function in PDAC cells was associated with decreased chemoresistance and survival *in vitro*[26,27]. In this experiment, SMAD4*-*proficient and -deficient PDAC cells were treated with three different kinds of chemotherapy drugs: cisplatin (Cis; 5 μM), gemcitabine, (Gem; 2 μM) and paclitaxol (Pac; 1 μM). Cells were seeded into 96-well plates in triplicate, treated with one of the chemotherapy drugs for 3 days, then analyzed by MTT assay, a commonly-used assay to measure cell viability after different chemotherapy drug treatments. Cell survival rates were measured to compare the SMAD4-positive and -negative groups in responding to different chemotherapy agents, and our *in vitro* data showed that the inactivation of SMAD4 may contribute to an increase in chemo-sensitivity in PDAC to different chemotherapy drugs (Figure 5 and Additional file 3: Figure S3).

**Figure 6:**
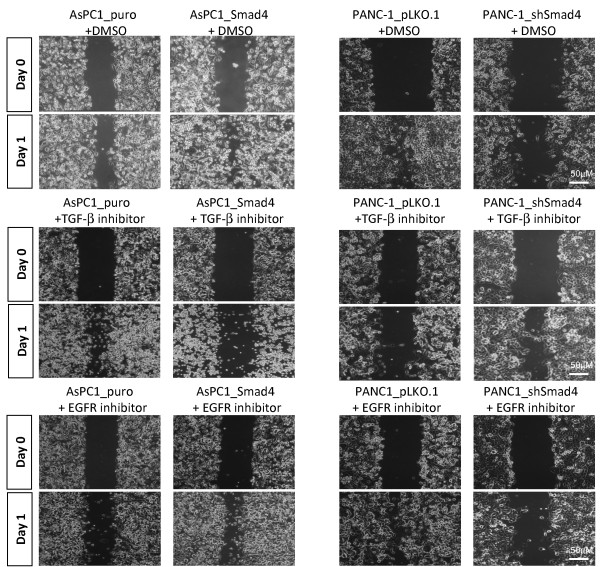
**SMAD4 proficient PDAC cells were more likely to respond to TGF-β1 inhibitor treatment, but SMAD4 deficient PDAC cells were more sensitive towards EGFR inhibitor treatment to block cell migration in vitro.** AsPC-1 and PANC-1 SMAD4 deficient and proficient cells were treated with TGF-β1 inhibitor SB231542 (0.5 μM) or EGFR inhibitor gefitinib (0.5 μM) and subjected to in vitro wound-healing assay. Each monolayer was scratched and incubated for overnight. The closure rate was photographed to compare their migratory ability between SMAD4 deficient and proficient PDAC cells with or without TGF-β1 or EGFR inhibitors treatment. Images are representative of three independent experiments (magnification 40×). P < 0.05.

In addition, many studies indicate that the TGF-β1 and EGFR signaling pathways are frequently activated during pancreatic carcinogenesis, and they have been shown to be crucial in promoting tumor cell migration and invasion [28,29]. We therefore investigated the relationship between SMAD4 status and cell migration in PDAC induced by the TGF-β1 and EGFR pathways. To investigate the specific effect of these two inhibitors on PDAC cellular migration independent of their proapoptotic effects *in vitro*, we first tested the IC50 values of each compound and applied a dose 5-fold below the IC50 value in order to eliminate any cytotoxic effect on proliferation and observe the drug’s anti-migration function *in vitro* (Additional file 4: Figure S4). We investigated whether inactivation of TGF-β1 by SB inhibitor 431542 suppresses the motility of SMAD4-positive or -negative PDAC cells *in vitro*. As shown in Figure 6, treatment of SMAD4-re-expressing AsPC-1 cells with 0.5 μM SB431542 caused a dramatic reduction in migration, but had no effect on these processes in SMAD4-null AsPC-1 control cells. Further, to evaluate whether inhibition of EGFR signaling can inhibit PDAC cell migration *in vitro*, wound healing assays were applied to SMAD4-positive and -negative PDAC cells after administration of 0.5 μM gefitinib, an EGFR tyrosine kinase inhibitor. The results showed that gefitinib treatment did not reduce cell migration of SMAD4-positive PDAC cells. In contrast, SMAD4-negative PDAC cells with high levels of EGFR expression exhibited significantly reduced cell motility when also exposed to gefitinib (P < 0.05; Figure 6 and Additional file 5: Figure S5). The same results were obtained by treating SB 431542 and gefitinib in PANC-1 shSMAD4 and pLKO.1 control cells (Figure 6). Our results imply that the efficacy of gefitinib treatment of PDAC cells is likely dependent on the cells’ EGFR activation status and, in particular, the loss of SMAD4. Notably, wound healing assays revealed the comparable and statistically significant (p < 0.05) ability of TGF-β and EGFR inhibitors to impede cell migration in our cell culture assays (Additional file 5: Figure S5).

## Discussion

SMAD4, also known as deleted in pancreatic carcinoma, locus 4 (DPC4), was first identified on the basis of frequent homozygous deletions and mutations affecting 18q21.1 in the pancreatic tumor, and was found to be involved in the TGF-β1 signaling pathway [11,30]. Germline mutations in SMAD4/DPC4 have also been identified in certain types of juvenile polyposis [31,32]. Hahn and colleagues reported that about 90 percent of pancreatic carcinomas show allelic loss at chromosome 18q21.1, and further studies have confirmed that the SMAD4/DPC4 gene, localized to 18q21, was the target for 50% of the PDAC that exhibited 18q deletion [12]. During carcinogenesis, TGF-β1 may act in an autocrine and/or paracrine fashion to exert a biphasic effect on cancer progression. Early in tumor formation, TGF-β1 functions to suppress cell cycle progression and block tumor growth. In contrast, cancer cells later adapt to develop a resistance to TGF-β1-mediated growth inhibition by increasing expression of TGF-β1 antagonist, mutating the TGF-β1 receptor or inactivating the SMAD4 gene. Subsequently, TGF-β1 ceases to function in tumor suppression and switches to the converse role of enhancing tumor metastasis by promoting tumor cells’ epithelial-mesenchymal transition (EMT) or inducing the angiogenic phenotype [33,34]. TGF-β1 is known to transduce signaling cascades through SMAD-dependent, as well as SMAD-independent, non-canonical pathways. A number of studies have reported that TGF-β1 can activate non-canonical SMAD-independent pathways through Ras/Erk (p44/42), PI3K/Akt, JNK or TAK1/p38 kinase [35,36]. However, the overall effect of Erk, Akt or p38 MAPK activation by TGF-β and the biological consequences are poorly characterized. Upon SMAD4 inactivation or deletion, TGF-β1 may preferentially signal through a SMAD-independent pathway, instead of the canonical SMAD-dependent pathway, leading to the phenotypic changes seen in tumor cells.

The study reported by Dai et al. [37] revealed that he antitumor activity of SMAD4 induces G1 arrest and apoptosis through the nuclear translocation of SMAD4 in MDAMB468 breast cancer cells, revealing the anti-tumor proliferation mediation of SMAD4-dependent signaling. Although most attention has focused on the cell cycle arrest mediated by TGF-β1/SMAD4 signaling, the other tumor suppressive effects of SMAD4 in preventing late stage tumor progression are still not fully understood. Until recently, our group and others have found SMAD4 involved in suppression of metastasis, angiogenesis and chemo-resistance in many different types of cancers [21,38]. For example, Schwarte- Waldhoff and his colleagues reported that the restoration of SMAD4 in SW480 colon cells reduced expression levels of the endogenous urokinase-type plasminogen activator and plasminogen-activator-inhibitor-1 (PAI-1) genes, involved in the degradation of extracellular matrix proteins and the control of tumor cell migration and invasion [39]. In 2000, they further demonstrated that SMAD4 re-expression in the human PDAC cell line Hs766T suppresses angiogenesis through down-regulation of VEGF and up-regulation of throbospondin-1 (TSP-1), a potent endogenous angiogenesis inhibitor [25]. Recently, our research group also reported that SMAD4 suppresses the development of malignant phenotypes of human colorectal cancer through interacting with HIF1α to suppress VEGF and MMP expression under hypoxic conditions [21]. Although these studies provide promising evidence of the role of SMAD4 as a tumor suppressor gene, our mechanistic understanding of SMAD4 is still in its infancy.

In the present study, using human PDAC cell lines, we first examined the overall effects of the restoration and knockdown SMAD4 expression in human PDAC cells. Specifically, we found that all PDAC cells exhibit increased cell migration *in vitro* after SMAD4 re-expression, although PDAC cell growth was not significantly affected after SMAD4 reconstitution. In addition, we observed that SMAD4 deficiency in human PDAC cells induces E-cadherin expression and such cells exhibit epithelial morphology, a result consistent with our previous report with SMAD4-conditional knockout mice demonstrating that genetically engineered mouse (GEM) models of Pdx Kras Smad4^L/L^ Ink/Arf^L/+^ mice develop more well-differentiated lesions with glandular structures of PDAC tumors than SMAD4 wild type Pdx Kras Ink/Arf^L/+^ mice [8]. Here, we also demonstrated an increase in the noncanonical or non-SMAD TGF-β pathways, including the MEK/ERK and PI3K/Akt signaling pathways, in SMAD4-negative PDAC cells compared to SMAD4-positive PDAC cells. Intriguingly, we also observed the down-regulated PTEN gene expression in SMAD4-deficient PDAC cells, an effect which may be partly due to the mediation of the inhibitory effects of NF-κB activation [40]. Previous studies have shown that TGF-β-activated kinase 1 (TAK1) is implicated in p38 MAPK activation in response to TGF-β1 in several cell systems [41]. In addition, TGF-β-induced EMT was blocked by inhibiting the activation of p38 MAPK in mouse mammary epithelial cells, and p38 MAPK inhibitors blocked TGF-β1-stimulated migration of non-tumor and tumor cells, which suggest that p38 MAPK may act in parallel or in cooperation with a SMAD-dependent pathway in chemotactic responses to TGF-β1 [42,43]. In this study, we also observed an increased activation of the p38 MAPK pathway in the presence of SMAD4 in PDAC. In addition, our result revealed that restoration of SMAD4 induces the increased activation of p38 MAPK signaling, which may in turn enhance the expression of c-Jun, c-fos or Fast-1 transcriptional factors in PDAC [44,45].

**Figure 7:**
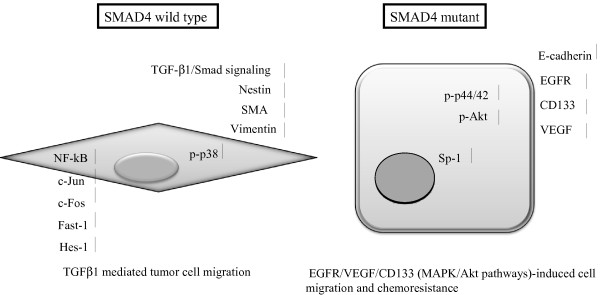
**A Model of phenotypic alteration involving SMAD4 loss in PDAC cells.** SMAD4 wild type PDAC cells exhibits more fibroblast-like morphology, which high express Nestin, SMA, CD44 gene, with the increase of activation of p38 MAPK and TGFβ1 signaling, and acquisition of a more high expression levels for the c-Jun, c-Fos, Hes1 NF-κb transcription factor genes. In contrast, inactivation of SMAD4 in PDAC cells exhibits more well differentiate epithelial like (cobblestone) morphology and leads to an overall increase in E-cadherin, CD133, VEGF, EGFR and Sp-1 expressions with high levels of activation p-44/42 and PI3K/Akt signaling pathways.

Most importantly, our present study provides the first experimental evidence that inactivation of SMAD4 enhances EGFR and CD133 expression, whereas re-expression of SMAD4 suppresses EGFR and CD133 levels in PDAC cells. These results are consistent with a previous report using HPDEC cells in which the knockdown of SMAD4 expression was found to increase EGFR expression [24]. Meanwhile, the down-regulation of EGFR expression in SMAD4-proficient cells may result from the reduced expression of the transcriptional factor Sp-1 (Figure 7). Recently, the CD133 molecule has been linked to tumor malignancy and invasiveness, and overexpression of EGFR and its ligands significantly contributes to the malignant phenotype and correlates with decreased survival in pancreatic cancer patients [46-49]. Further insight is needed to evaluate the relationship between the expression levels of EGFR and the presence of CD133 in PDAC, and the association between EGFR and CD133 may represent an important mechanism in the control of SMAD4- inactivated PDAC cell proliferation and malignancy. Our data further indicated increased Nestin expression upon SMAD4 reconstitution in PDAC, a result which may be related to the restoration of the TGF-β1/SMAD signaling pathway in PDAC cells. Nestin was first identified as an important neuronal stem cell marker during central nervous system development [50,51]. The long carboxy-terminal portion of Nestin has been reported to serve as the link or cross-bridge between intermediate filaments and microtubule, helping to mediate cell migration. Recently, Matsuda and colleagues illustrated the importance of Nestin in pancreatic cancer cell migration, invasion and metastasis by selectively modulating the expression of actin and other cell adhesion molecules [52]. They proposed that Nestin expression is crucial for colonizing distant sites in metastasis and thus may be a marker of metastasis-initiated “cancer stem cells”. How SMAD4 regulates Nestin expression in PDAC is not yet clear. The Nestin promoter does harbor several potential SMAD-binding sites, two SBE-related sequence 5′-CAGACA-3′-box at position -2067 and -566. Thus, it could exert control via transcriptional regulation. More recently, we proposed that increased Nestin expression could provide a positive feedback loop to induce TGF-β1/SMAD signaling by increasing the expression of TGF-β1 and TβR1a and TβR2 receptors [20]. Nestin is also involved in regulating the Wnt effector; the CD44 gene, a known putative cancer stem cell marker involved in mediating tumor cell metastasis [53]. Thus, this study provides the first evidence linking SMAD4 status and the expression patterns of CSC markers of PDAC.

We also demonstrated that reconstitution of SMAD4 in PDAC cells resulted in an increase in apoptotic death after treatment with cisplatin, gemcitabine, or paclitaxel when compared with SMAD4-deficient PDAC cells. This result is in agreement with our previously published work in the colorectal cancer model, which found that SMAD4 loss increased resistance to the chemotherapeutic agent 5′-fluorouracil [21]. Many more recent studies have shown that TGFβ1 and EGFR inhibitors are promising for the treatment of pancreatic cancer [54-56]. Like many chemotherapeutic agents, the effectiveness of EGFR inhibitors have been approved by Food and Drug Administration for use in several tumor cases, alone and in combination with gemcitabine for pancreatic cancer [57,58]. In the present study, we concluded that treatment of SMAD4-proficient PDAC cells with TGF-β1 inhibitor resulted in a profound reduction in cell migration *in vitro*. In contrast, treatment with EGFR inhibitor remarkably inhibited cell migration in SMAD4-deficient PDAC cells, implying that the SMAD4 defect results in a gain to the EGFR signaling pathway during PDAC development.

## Conclusions

The present study revealed the molecular basis for SMAD4-dependent and -independent differences in PDAC tumor biology with the aim of identifying the subset of patients likely to respond to therapies targeting the TGF-β or EGFR signaling pathways (Figure 7) The use of model system illustrated here may help to identify additional nodes of therapeutic intervention in PDAC patients devoid of SMAD4.

## Competing interests

No potential conflicts of interest were disclosed.

## Authors’ contributions

WYC AB and ES; PJH ES; CCW JY; KKK FG; TLK JY; DCW FG; WCH FG and KHC FG. All authors read and approve the final manuscript.

## Pre-publication history

The pre-publication history for this paper can be accessed here:

http://www.biomedcentral.com/1471-2407/14/181/prepub

## Supplementary Material

Additional file 1: Figure S1 SMAD4 enhances migration and invasiveness of AsPC-1 and PANC-1 cells in vitro. Representative images of the invaded cells are represented. Invading cells on the lower surface that passed through the filter were fixed and stained using crystal violet in gluteraldehyde and photographed. Scale bar, 50 μm. Click here for file

Additional file 2: Figure S2 Immunohistochemistry (IHC) analysis evaluates E-cadherin, EGFR, CD133, Nestin and SMAD4 expression levels in PANC-1 shSMAD4 and control xenograft tumors. Tumor sections were analyzed by H&E and IHC using anti-SMAD4, anti-Ecadherin, anti-CD133, anti-Nestin and anti-EGFR antibodies as described in Material and methods section. Tissues were stained with 3,3′- diaminobenzidine (brown) and counterstained with hematoxylin (blue). Scale bar, 50 μm. Click here for file

Additional file 3: Figure S3 Morphological characterization under phase contrast microscopy of cell death in SMAD4 proficient and deficient AsPC-1 and PANC-1 cells after different chemo drugs treatment. Bright field microscopy images are representative fields of the cell morphology of SMAD4 proficient or deficient cells were incubated in medium in the presence of DMSO, cisplatin (Cis), paclitaxel (Pac) or gemcitabine (Gem) treatment for 2 days. Scale bar, 50 μm. Click here for file

Additional file 4: Figure S4 Dose response of SMAD4 positive and negative PDAC cells to SB431542 and gefitinib. The cells were treated with various doses of SB431542 or gefitinib for 24 hours, and the cell viability was measured by a MTT assay. Data represent the mean values ± standard error of three independent experiments. Click here for file

Additional file 5: Figure S5 Quantitation of cell migratory ability in SMAD4 proficient and deficient AsPC-1 and PANC-1 cells after different inhibitor treatments. Wounded area per field was individually assessed and averaged per well. To determine scale, a picture was taken of a micrometer, and two to three fields on each filter were scored for cell migration under an inverted microscope. Calibration was performed with the analysis tool in Image J. Data represent relative cell migration ability normalized to vector control cells treated with DMSO (mean ± SD, n = 3; combined data from two independent experiments each performed in triplicate). Significantly different (*P < 0.05) compared with different conditions. Click here for file
