# Supplementary figures and images for: SMAD4 Loss triggers the phenotypic changes of pancreatic ductal adenocarcinoma cells
Source: BMC Cancer. 2014 Mar 14;14:181. doi: 10.1186/1471-2407-14-181 (PMC4007528; doi:10.1186/1471-2407-14-181)

Supplementary Figure 1

**AsPC-1**

puro

SMAD4

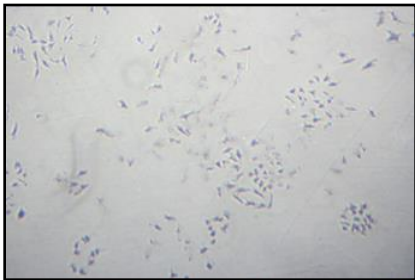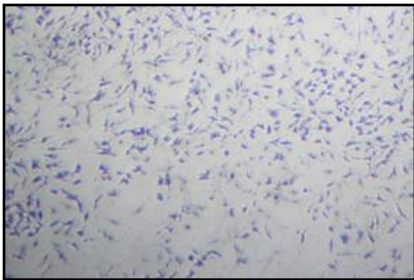

**PANC-1**

pLKO.1

shSMAD4

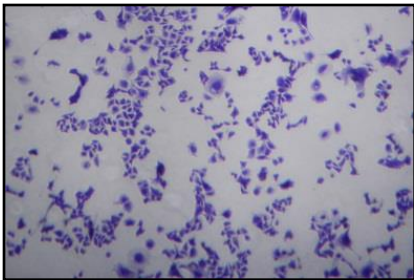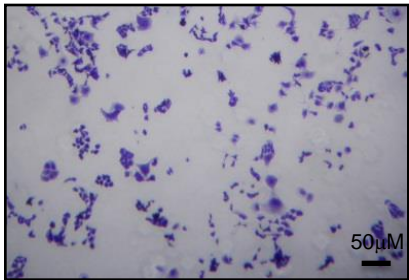

Supplement: Additional file 1: Figure S1 — SMAD4 enhances migration and invasiveness of AsPC-1 and PANC-1 cells in vitro. Representative images of the invaded cells are represented. Invading cells on the lower surface that passed through the filter were fixed and stained using crystal violet in gluteraldehyde and photographed. Scale bar, 50 μm. [file 1471-2407-14-181-S1.pdf]

Supplementary Figure 2

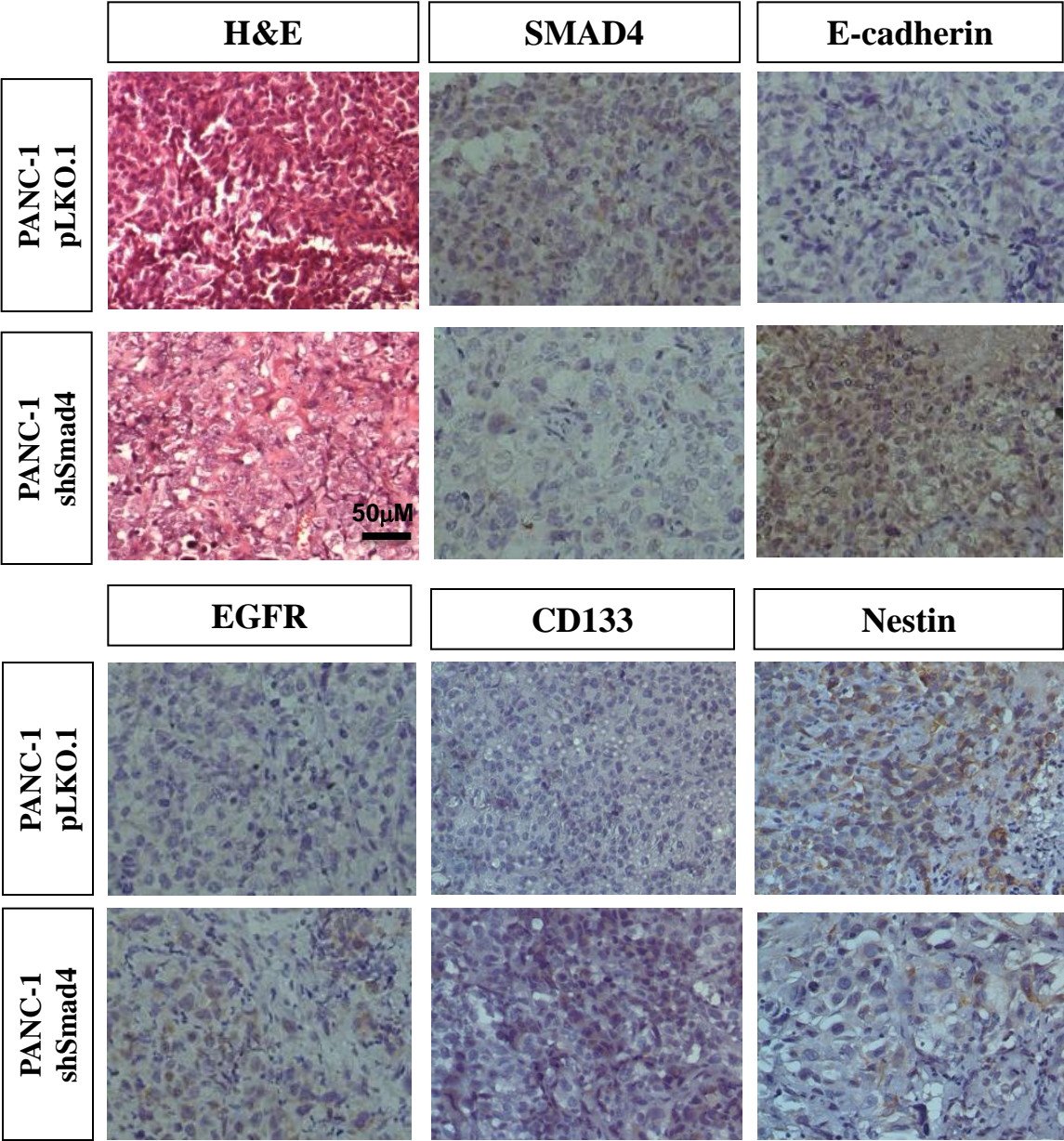

Supplement: Additional file 2: Figure S2 — Immunohistochemistry (IHC) analysis evaluates E-cadherin, EGFR, CD133, Nestin and SMAD4 expression levels in PANC-1 shSMAD4 and control xenograft tumors. Tumor sections were analyzed by H&E and IHC using anti-SMAD4, anti-Ecadherin, anti-CD133, anti-Nestin and anti-EGFR antibodies as described in Material and methods section. Tissues were stained with 3,3′- diaminobenzidine (brown) and counterstained with hematoxylin (blue). Scale bar, 50 μm. [file 1471-2407-14-181-S2.pdf]

Supplementary Figure 3

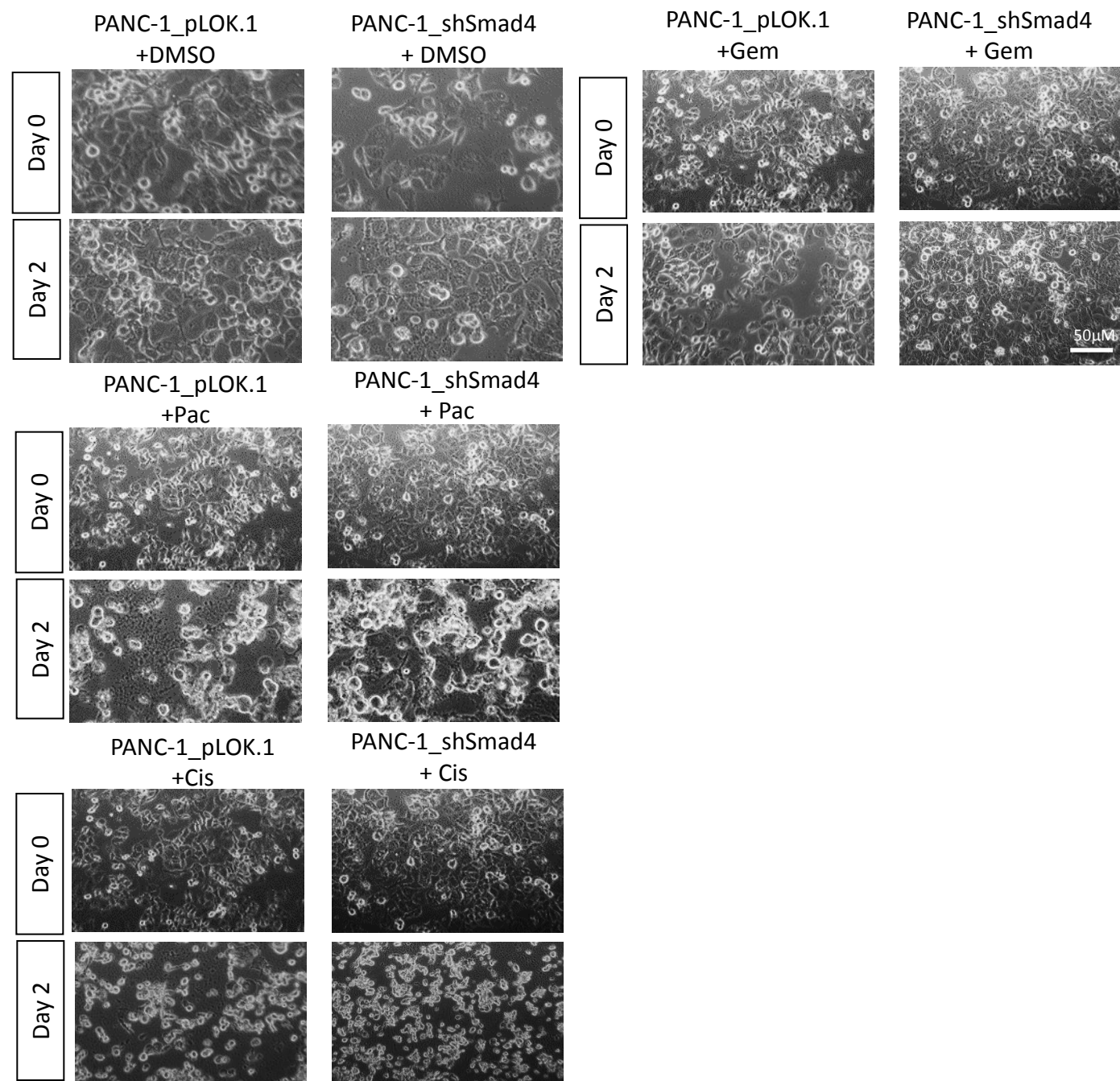

Supplement: Additional file 3: Figure S3 — Morphological characterization under phase contrast microscopy of cell death in SMAD4 proficient and deficient AsPC-1 and PANC-1 cells after different chemo drugs treatment. Bright field microscopy images are representative fields of the cell morphology of SMAD4 proficient or deficient cells were incubated in medium in the presence of DMSO, cisplatin (Cis), paclitaxel (Pac) or gemcitabine (Gem) treatment for 2 days. Scale bar, 50 μm. [file 1471-2407-14-181-S3.pdf]

Supplementary Figure 4

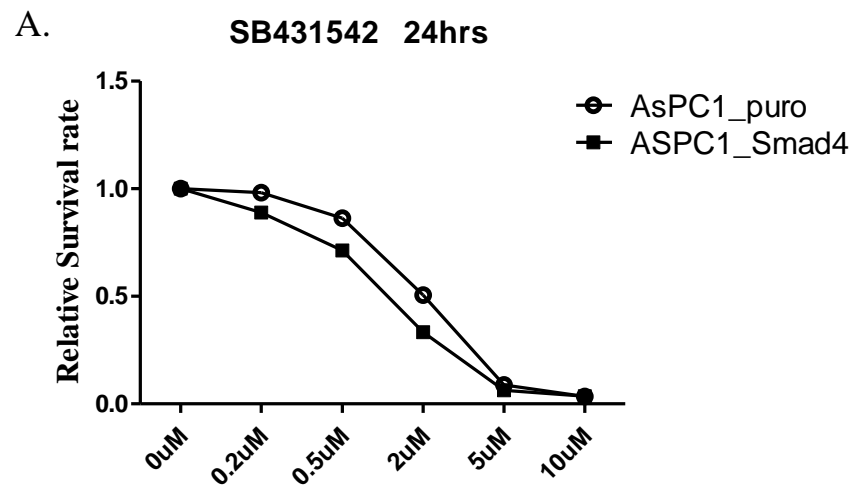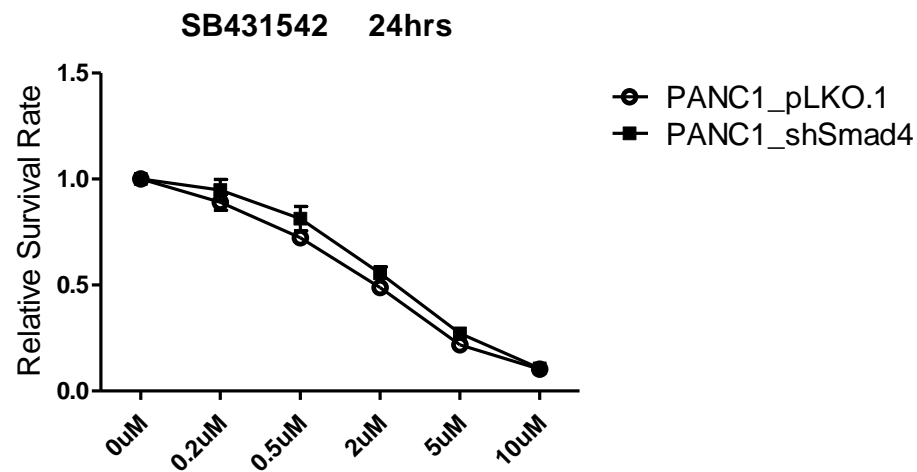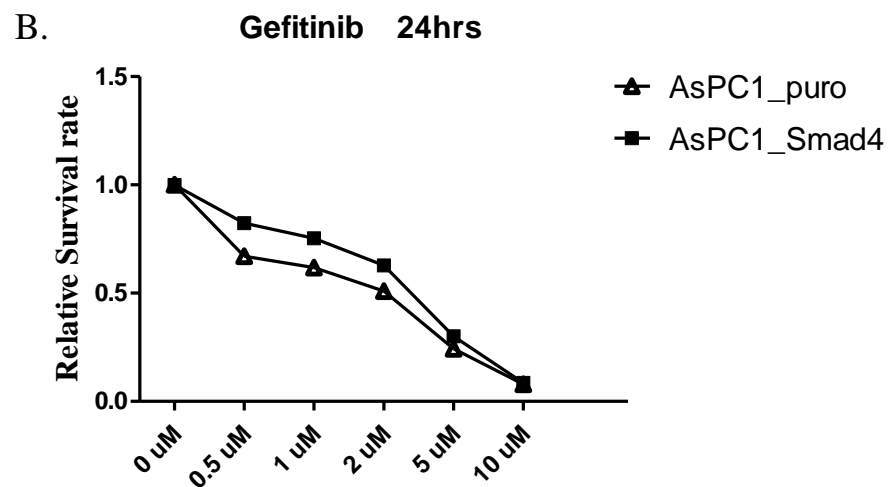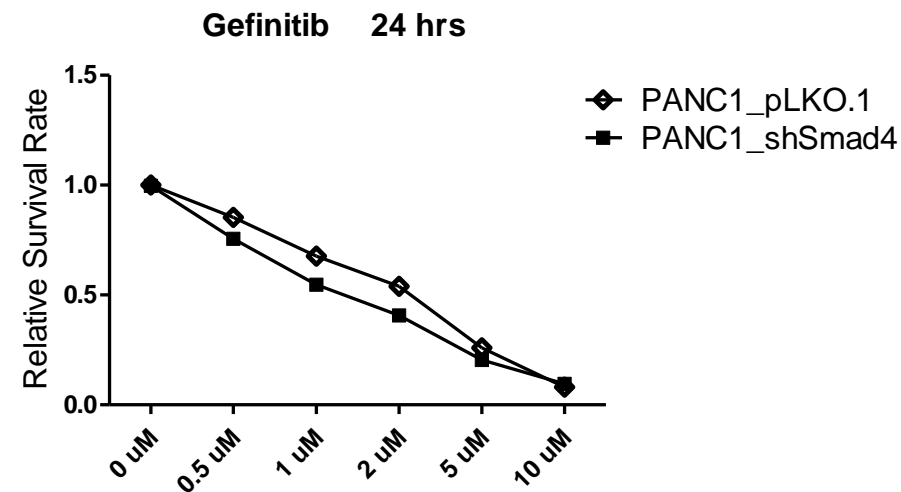

Supplement: Additional file 4: Figure S4 — Dose response of SMAD4 positive and negative PDAC cells to SB431542 and gefitinib. The cells were treated with various doses of SB431542 or gefitinib for 24 hours, and the cell viability was measured by a MTT assay. Data represent the mean values ± standard error of three independent experiments. [file 1471-2407-14-181-S4.pdf]

Supplementary Figure 5

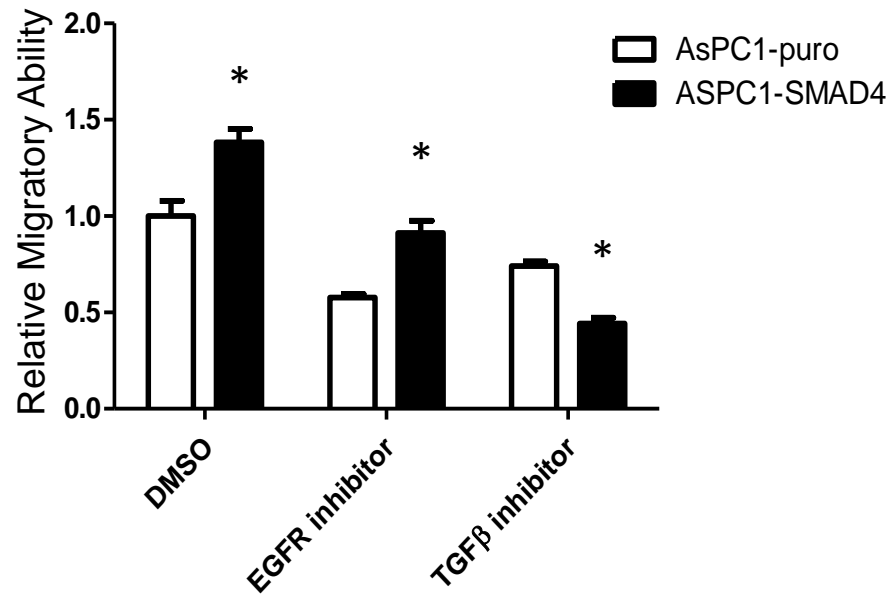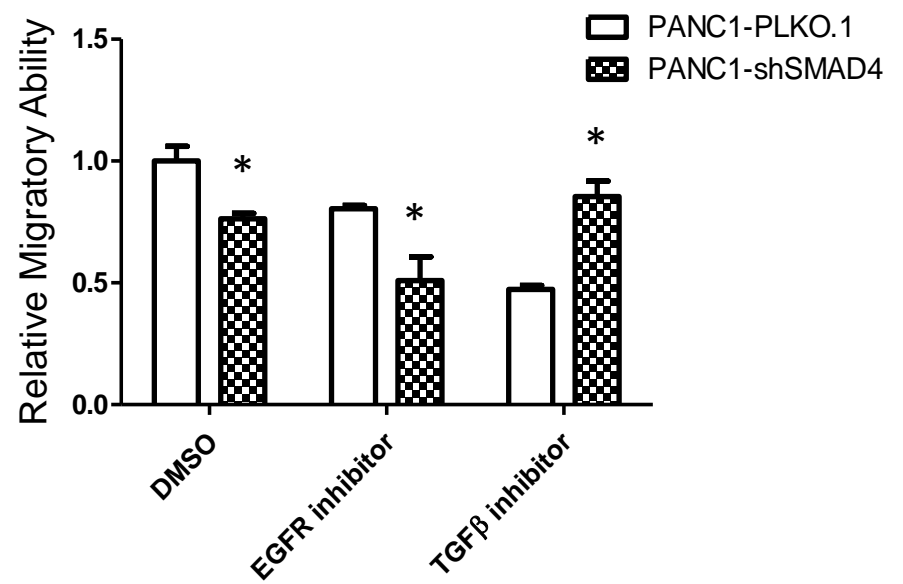

Supplement: Additional file 5: Figure S5 — Quantitation of cell migratory ability in SMAD4 proficient and deficient AsPC-1 and PANC-1 cells after different inhibitor treatments. Wounded area per field was individually assessed and averaged per well. To determine scale, a picture was taken of a micrometer, and two to three fields on each filter were scored for cell migration under an inverted microscope. Calibration was performed with the analysis tool in Image J. Data represent relative cell migration ability normalized to vector control cells treated with DMSO (mean ± SD, n = 3; combined data from two independent experiments each performed in triplicate). Significantly different (*P < 0.05) compared with different conditions. [file 1471-2407-14-181-S5.pdf]
